# Effects of Lutein Supplementation in Japanese Patients with Unilateral Age-Related Macular Degeneration: The Sakai Lutein Study

**DOI:** 10.1038/s41598-020-62483-0

**Published:** 2020-04-06

**Authors:** Miki Sawa, Takuya Shunto, Issei Nishiyama, Ayako Yokoyama, Ryujiro Shigeta, Satoko Miura, Ryo Kawasaki

**Affiliations:** 1 Eye Center, Sakai City Medical Center, Osaka, Japan; 2 Department of Ophthalmology, Osaka Habikino Medical Center, Osaka, Japan; 3 Department of Vision Informatics, Osaka University Graduate School of Medicine, Osaka, Japan

**Keywords:** Medical research, Risk factors

## Abstract

This prospective randomized double-masked study investigated the effects of 20 mg lutein supplementation with two different capsules (beeswax or glycerol fatty acid esters) for 6 months on the fellow eyes of 39 Japanese patients with unilateral age-related macular degeneration, and assessed the factors associated with baseline plasma lutein concentration via lifestyle interviews. Macular pigment optical density (MPOD), determined with the two-wavelength autofluorescence method, increased over time in the beeswax group (ANOVA, p = 0.0451), although the increase from 3 months to 6 months was only marginally significant. No significant increase was observed in the glycerol fatty acid esters group (ANOVA, p = 0.7396). Plasma lutein concentrations significantly increased at 3 and 6 months from baseline in both groups (both p < 0.01). In a multiple regression model, age was negatively associated with higher plasma lutein concentration (p = 0.0305), while consumption of green vegetables was positively associated with baseline plasma lutein concentration (p = 0.0322). In conclusion, a significant increase in MPOD was not fully confirmed with 6 months intake duration despite a significant increase in plasma lutein concentrations. Consumption of green vegetable was confirmed to be associated with plasma lutein concentration after adjusting for other potential factors including age.

## Introduction

Age-related macular degeneration (AMD) is the leading cause of blindness worldwide in elderly individuals. Epidemiological studies and gene research have revealed that aging, genetics, and smoking are associated with AMD development^1^. Oxidative stress from continuous light exposure has been implicated in the aetiology and pathogenesis of AMD^2^. Thus, antioxidant supplementation can reduce oxidative stress, and it is thought to be one strategy to prevent AMD development^2^.

The Age-Related Macular Eye Disease Study (AREDS) demonstrated that supplementation with antioxidant vitamins and minerals reduced the risk of development of advanced AMD by 25% at 5 years^3^. AREDS2 described the efficacy of switching to lutein/zeaxanthin from beta-carotene as an antioxidant, although additional DHA/EPA did not show benefits over the AREDS formula at the 5-year follow-up assessment^4^. Interestingly, in the AREDS2 sub-analyses, antioxidant supplementation for the patients in the lowest vegetable intake quantile reduced the risk of development to advanced AMD by 26%^4^. Vegetable intake and blood lutein concentration have also been suggested to be correlated in other reports^5–7^. Thus, oral lutein supplementation is beneficial for patients with suspected low blood lutein levels, and investigating the factors associated with blood lutein level is expected to be important in preventing AMD development.

Various methods have been used to examine the efficacy of lutein supplementation, including measurement of the rate of AMD development, which was used in the AREDS study, blood lutein monitoring, contrast sensitivity assessment, and macular pigment optical density measurement^8–10^. Among these, the latter three are quantitative assessment methods performed over relatively short periods of time. Obana *et al*. performed some lutein supplementation studies in Japanese subjects by monitoring their blood lutein levels and evaluating contrast sensitivity and macular pigment optical density^8,11^. Their results revealed the existence of non-responders to lutein supplementation among healthy young Japanese volunteers^11^. Thus, similar non-responders to lutein supplementation can be expected among older AMD patients because lutein supplements are absorbed in the intestine, and absorption from the gastrointestinal tract may be poorer in elderly AMD patients. In such scenarios, modification of the soft capsule material is one option to increase the absorption of lutein in the intestine. For many decades, beeswax and glycerol fatty acid esters have been commonly used for the preparation of soft capsules for various supplements as a dispersant^12^. Recently, products using glycerol fatty acid esters have been improved to have better dissolution^13,14^. To the best of our knowledge, no study to date has evaluated the effect of the soft capsule material on the absorption and availability of lutein.

We conducted a lutein supplementation study for subjects with unilateral AMD by comparing two types of soft capsule materials. In addition, factors associated with baseline plasma lutein concentration, including age, sex, AMD subtype of the fellow eye, hypertension, smoking, and frequency of green vegetable intake were also investigated in the current study.

## Subjects and Methods

This was a prospective, parallel-group comparison, double-masked study conducted at a single institution (Eye Center, Sakai City Medical Center, Osaka, Japan). Following the Ethical Guidelines for Medical and Health Research Involving Human Subjects by the Ministry of Health, Labor and Welfare, Japan, this study was performed after being approved by the Institutional Review Board of Sakai City Medical Center (Sakai City, Osaka, Japan) on September 6, 2016 (No. 20). This trial study (originally, randomized parallel-group trial of lutein supplementation for macular pigment optical density and visual function in the patients with unilateral age-related macular degeneration) was registered as No. UMIN000027962 (June 28, 2017).

### Subjects

Patients with unilateral exudative AMD who required periodic examinations and anti-vascular endothelial growth factor (VEGF) therapy and/or photodynamic therapy were recruited at our institute. The AMD subtype (typical AMD or polypoidal choroidal vasculopathy, PCV) was determined by fluorescein and indocyanine angiography before treatment. The patients received a full explanation of the study and signed an informed consent form in compliance with the tenets of the Declaration of Helsinki.

Before the enrolment, all subjects were asked regarding their general condition in relation to diabetes mellitus, hypertension, hyperlipidaemia, and cancer. The inclusion criteria were the presence of unilateral AMD and a visual acuity equal to or more than 0.8 in the fellow eye. The subject eyes of this study were the fellow eyes showing normal findings or early age-related maculopathy (ARM) in optical coherence tomography and angiography. The criteria for early ARM were the presence of small soft drusen, hard drusen, and retinal pigment epithelium (RPE) abnormalities including depigmentation or pigmentation on the basis of an international classification system for the definition of early ARM^15^. Eyes with mild cataract (>grade 2, Emely–Little classification) were excluded because of the associated disturbances in rendering valid measurements. Eyes that had undergone cataract surgery at least 3 months prior to enrolment were eligible. Patients with a 3-month history of regular intake of lutein and/or zeaxanthin and corticosteroid treatment were excluded. If patients who had a history of regular lutein supplement intake wished to join this study, their enrolment was allowed three months after discontinuation of lutein supplementation. Eyes with other retinal disorders such as retinal vein occlusion and apparent diabetic retinopathy were also excluded.

### Lutein supplement

A lutein supplement (Sante Lutax® 20, Santen Pharmaceutical, Osaka, Japan) containing 20 mg lutein and 3 mg zeaxanthin in two different capsule types (beeswax or glycerol fatty acid esters as a dispersant) was used in this study. The Sante Lutax® 20 with beeswax capsule was commercially available in Japan before 2015. However, in 2015, the soft capsule material used in Sante Lutax® 20 was changed from beeswax to the glycerol fatty acid esters (EMAX® BW-36, Riken Vitamin, Tokyo, Japan) used in the crocetin supplement^16^. In this study, we compared the two types of Sante Lutax® 20 capsules, prepared with either beeswax or glycerol fatty acid esters.

### Study design

This was a prospective, parallel-group comparison, double-masked study conducted at a single institution from October 2015 to December 2017. The beeswax and glycerol fatty acid esters capsule groups were randomized 1:1 using the block randomization method. The subjects were sequentially assigned by the investigator. The numbered packages containing lutein supplement tablets (beeswax or glycerol fatty acid esters capsule) were prepared by the sponsor company. The investigators were blinded to the allocation until the end of the study. The duration of lutein intake (one tablet in a day) was 6 months. The required number of subjects was determined based on the following: a desired power of 0.80 and a significant difference between groups of 0.09 in the change in MPOD, with σ = 0.1. The resulting sample size was calculated as 41.

### Outcome measurements

Periodic examinations were performed once every three months (baseline, 3, and 6 months). The primary measurement outcome was the difference in the macular pigment optical density (MPOD) between baseline and 6 months, which was determined using the two-wavelength fundus autofluorescence method. The secondary outcomes were the differences in plasma lutein concentration and contrast sensitivity between baseline and 6 months. At the final visit, an interview was conducted to assess patient lifestyle.

### MPOD measurement

Before macular pigment evaluations, the pupils were dilated with 0.5% tropicamide and 2.5% phenylephrine eye drops, and MPOD was determined by autofluorescence spectrometry with two wavelengths (488- and 514-nm excitation wavelengths and a band-pass filter at a wavelength of 530 nm), as described previously^17^. The instrument (Heidelberg Retina Angiograph, Heidelberg, Germany) was the same as that used in previous lutein supplementation studies^18,19^. The system software creates an averaged autofluorescence image from two videos consisting of 16 images. Macular pigment absorption is higher at 488 nm than at 514 nm; thus, subtraction of the autofluorescence signals between these two images provides the MPOD map in the central retina. Data were excluded if they showed a decrease in the number of effective pixels (<150/225 pixels) due to poor fixation. The density was expressed in optical density units (DU). We used the mean optical density as the MPOD within a 1-degree diameter circle centred on the fovea.

### Plasma lutein concentration

Blood samples were obtained at baseline and 3 and 6 months. The plasma lutein concentration was measured using a high-performance liquid chromatography (HPLC) system (LC-2010C; Shimadzu Corp., Kyoto, Japan) in Sumika Chemical Analysis Service, LTD (Osaka, Japan). Plasma samples were extracted with a mixture of methyl tertiary butyl ether (4:1) and centrifuged at 3,000 rotations/minute for 5 min at 4 °C. After evaporation of the organic layer under a nitrogen gas stream, the residue was dissolved in a 0.2-mL mobile phase solution for HPLC injection. The column was a GL Science Inertsil ODS-3 (5-µm particle size, 4.6-mm inside diameter × 250 mm). The column temperature was 40 °C. A mobile phase containing acetonitrile, 0.1 M ammonium acetate and methanol, and dichloromethane (71:22:7, v/v/v) was used. The flow rate was 1.0 mL/min. The 450-nm wavelength was used.

### Contrast sensitivity

Contrast and glare sensitivity were measured using glare-testers (Model CGT-2000, Takagi, Nagano, Japan). Contrast threshold values were measured at six visual angles (sizes) of the target (6.3, 4.0, 2.5, 1.6, 1.0, and 0.64 degrees) under a mesopic background (10 cd/m^2^), and the threshold values under a glare background (10,000 cd/m^2^) were measured with the same target sizes. The area under log contrast sensitivity function (AULCSF) was calculated using Excel software, and contrast sensitivity was statistically analysed using AULCSF^20^.

### Lifestyle interviews

During the final assessment, performed after 6 months, we interviewed the patients to determine lifestyle-related factors, including smoking and food habits. The questionnaire evaluation for smoking (never, past, or current) was performed as follows: if the patient had a smoking history, we interviewed the number of cigarettes smoked per day and the number of years and then calculated the smoking index (the number of cigarettes smoked per day by number of years) in past and current smokers.

A questionnaire evaluation regarding food habits and the frequency of intake of green vegetables such as spinach, leaf lettuce, and broccoli was also performed. The frequency was scored from 0 (none in a week) to 6 (equal to or more than  two times in a day), as shown in Table 1.

**Table 1:** Scoring system for the food intake frequency questionnaire.

Score	Frequency of intake
0	Never in a week
1	Less than one time in a week
2	One time in a week
3	2–3 times in a week
4	4–6 times in a week
5	One time in a day
6	>=Two times in a day

### Data analysis

Statistical analysis was performed using the JMP version 14 software (SAS Institute, Cary, NC). Mean changes in MPOD, plasma lutein concentration, and AULCSF from the baseline to 3 months and 6 months were analysed by repeated measures analysis of variance (ANOVA). Pair-wise comparisons were conducted with t-test with Bonferroni’s correction as post hoc analyses. A p-value (p) < 0.05 was considered statistically significant. The differences between beeswax capsule and glycerol fatty acid esters capsule groups over time for MPOD and plasma lutein concentration were analysed by analysis of covariance (ANCOVA). Factors associated with baseline plasma lutein concentration were compared between those treated with lutein supplement with beeswax or glycerol fatty acid esters capsule by t-test, followed by a multiple regression analysis.

## Results

Forty-two patients (25 men and 17 women) with unilateral AMD were recruited from October 2016 to June 2017. Three patients were excluded from the final analysis for the following reasons: one patient discontinued periodic examinations due to bone fracture; one patient took three tablets in a day (overdosing); and one patient had yellow skin one month after the lutein supplement intake.

The data from 39 patients were finally analysed in this study, and the demographic data at baseline are shown in Table 2. In all 39 patients, the number of remaining capsules was assessed at every examination, and compliances were confirmed during the study. As shown in Table 2, baseline parameters did not significantly differ between the beeswax capsule and glycerol fatty acid esters capsule groups except for the proportion of patients with phakic or intraocular lenses.

**Table 2:** Demographic data at baseline.

	Total	Beeswax capsule group	Glycerol fatty acid esters capsule group	P Value
No. of eyes	39	20	19	
Age, mean ± SD (years)	70.7 ± 5.3	69.5 ± 5.6	72.0 ± 4.8	0.1359^*a^
Male/female	24/15	13/7	11/8	0.6485^*b^
BMI, mean ± SD	23.3 ± 3.2	22.8 ± 2.4	23.8 ± 3.8	0.3475^*a^
Smoking history, current/past/never	4/21/14	3/9/8	1/12/6	0.4296^*b^
Hypertension (Y/N)	19/20	11/9	8/11	0.4207^*b^
Diabetes Mellitus (Y/N)	5/34	2/18	3/16	0.5888^*b^
Hyperlipidaemia (Y/N)	8/31	6/14	2/17	0.1322^*b^
Phakia/IOL	30/9	18/2	12/7	0.0467^*b^
Typical AMD/PCV	15/24	5/15	10/9	0.0762^*b^

SD = standard deviation; BMI = body mass index; Y/N = Yes/No; IOL = intraocular lens; AMD = age-related macular degeneration; PCV = polypoidal choroidal vasculopathy.

*^a^unpaired t-test, *^b^Pearson’s chi-squared test.

All patients had a history of intravitreal aflibercept injection, and 15 patients had photodynamic therapy for PCV eyes in our institute. When the study started, 16 patients were in the loading phase of anti-VEGF therapy and 23 patients were in the maintenance phase by pro re nata. During the study, 24 eyes were treated with intravitreal aflibercept injection; 15 eyes did not undergo this treatment.

### Changes in MPOD

**Figure 1:**
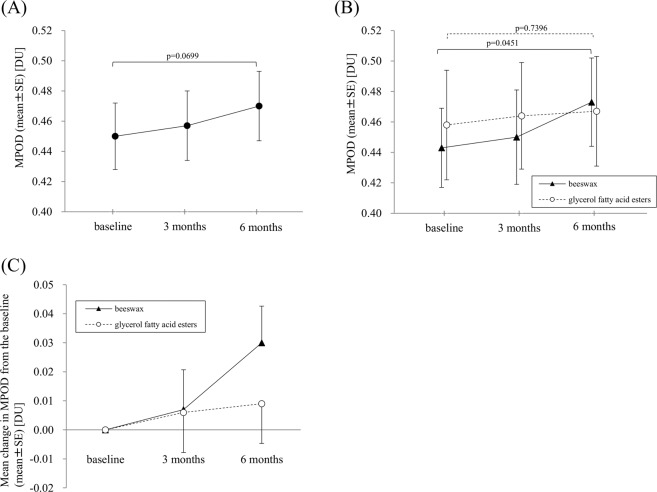
Changes in macular pigment optical density (MPOD) values in a total of 39 fellow eyes of unilateral age-related macular degeneration patients (**A**) and the beeswax capsule group (n = 20) and glycerol fatty acid esters capsule group (n = 19) (**B**). Comparison of MPOD difference from the baseline in the beeswax capsule group (n = 20) and glycerol fatty acid esters capsule group (n = 19) (**C**). (**A**) MPOD gradually increased from the baseline over 3 and 6 months without significance (repeated measures ANOVA, p = 0.0699). (**B**) In the beeswax capsule group, MPOD marginally increased from the baseline (ANOVA, p = 0.0451; Bonferroni corrected pair-wise comparison between 3 months and 6 months, p = 0.0555). MPOD in the glycerol fatty acid esters capsule group did not significantly increase from the baseline (repeated measures ANOVA, p = 0.7396). (**C**) No significant differences from the baseline were seen between the beeswax capsule and glycerol fatty acid esters capsule groups at 3 and 6 months (ANCOVA; p = 0.9722 and p = 0.2891, respectively). MPOD = macular pigment optical density; DU = density units; SE = standard error.

The mean MPOD (±standard error, SE) was 0.450 ± 0.022 DU at baseline in all 39 subjects’ eyes. After lutein intake, the mean MPOD ± SE was 0.457 ± 0.023 DU at 3 months and 0.470 ± 0.023 DU at 6 months. MPOD gradually increased with time; however, it did not significantly increase from the baseline to 6 months (p = 0.0699) (Fig. 1A). In a comparison of the two capsule groups, the mean MPOD at baseline was 0.443 ± 0.026 DU in the beeswax capsule group and 0.458 ± 0.036 DU in the glycerol fatty acid esters capsule group (p = 0.7372). The changes in MPOD in each capsule group are shown in Fig. 1B. In the beeswax capsule group, there was a marginally significant increase in MPOD at 3 months to 6 months from the baseline (ANOVA, p = 0.0451; Bonferroni corrected pair-wise comparison between 3 months and 6 months, p = 0.0555). There was no significant increase in the MPOD between baseline and 6 months in the glycerol fatty acid esters capsule group (ANOVA, p = 0.7396). In the beeswax capsule and glycerol fatty acid esters capsule groups, the mean differences in MPOD from the baseline at 3 months were 0.007 DU and 0.006 DU, respectively, and those at 6 months were 0.030 DU and 0.009 DU, respectively (Fig. 1C). The intergroup differences were not significant at 3 nor 6 months (p = 0.9722 and p = 0.2891, respectively).

### Changes in plasma lutein concentrations

**Figure 2:**
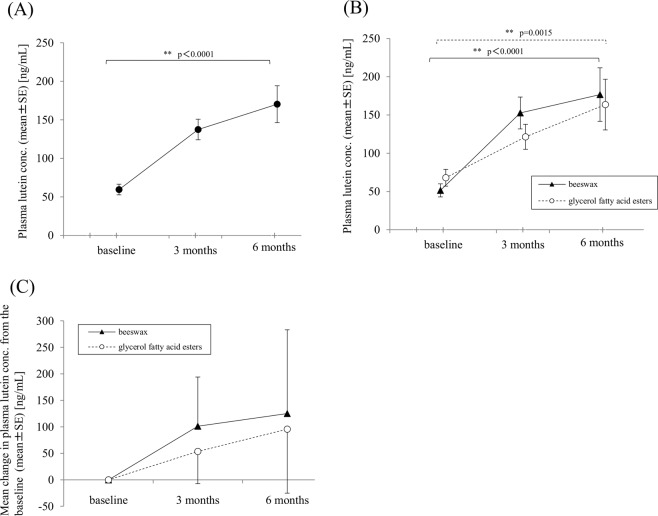
Changes in plasma lutein concentration in a total of 39 unilateral age-related macular degeneration patients (**A**) and the beeswax capsule group (n = 20) and glycerol fatty acid esters capsule group (n = 19) (**B**). Comparison of difference from the baseline in the beeswax capsule group (n = 20) and glycerol fatty acid esters capsule group (n = 19) (**C**). (**A**) Plasma lutein concentration significantly increased from the baseline at 3 months and 6 months (repeated measures ANOVA, p < 0.0001). (**B**) The plasma lutein concentrations significantly increased at 3 and 6 months from the baseline in the beeswax capsule group (repeated measures ANOVA, p < 0.0001) and at 6 months from the baseline in the glycerol fatty acid esters capsule group (repeated measures ANOVA, p = 0.0015). (**C**) No significant differences were seen between the two capsule groups at 3 and 6 months (ANCOVA; p = 0.0668 and p = 0.5171, respectively). SE = standard error.

At baseline, the mean (±SE) plasma lutein concentration in all 39 patients was 59.5 ± 6.9 ng/mL (range, 4 to 169 ng/mL). After lutein intake, the mean plasma lutein concentrations (±SE) were 137.4 ± 13.4 ng/mL at 3 months and 170.3 ± 23.9 ng/mL at 6 months (Fig. 2A). Plasma lutein concentration significantly increased between baseline and 3 months, and baseline and 6 months (ANOVA, p < 0.0001; Bonferroni corrected pair-wise comparison between baseline and 3 months, and baseline and 6 months, were both p < 0.003).

In the comparison between two capsule materials of beeswax and glycerol fatty acid esters, the mean (±SE) plasma lutein concentrations were 51.4 ± 8.6 ng/mL in the beeswax capsule group and 67.9 ± 10.9 ng/mL in the glycerol fatty acid esters capsule group at baseline, with no significant intergroup difference (p = 0.2395). Changes in plasma lutein concentrations in both capsule groups are shown in Fig. 2B. The plasma lutein concentrations significantly increased at 3 months and 6 months from the baseline in the beeswax capsule group (ANOVA, p < 0.0001; Bonferroni corrected pair-wise comparison between baseline and 3 months, and baseline and 6 months, were p = 0.0003 and p = 0.0066, respectively) and the glycerol fatty acid esters capsule group (ANOVA, p = 0.0015; Bonferroni corrected pair-wise comparison between baseline and 3 months, and baseline and 6 months, were p = 0.0036 and p = 0.0087, respectively). The mean changes in plasma lutein concentration from the baseline in the beeswax capsule group and the glycerol fatty acid esters capsule group were 101.4 ng/mL and 53.5 ng/mL at 3 months and 125.2 ng/mL and 95.5 g/mL at 6 months, respectively (Fig. 2C). No significant differences were seen between the two capsule groups at 3 and 6 months (p = 0.0668 and 0.5171, respectively).

### Contrast sensitivity assessment using the glare tester

**Figure 3:**
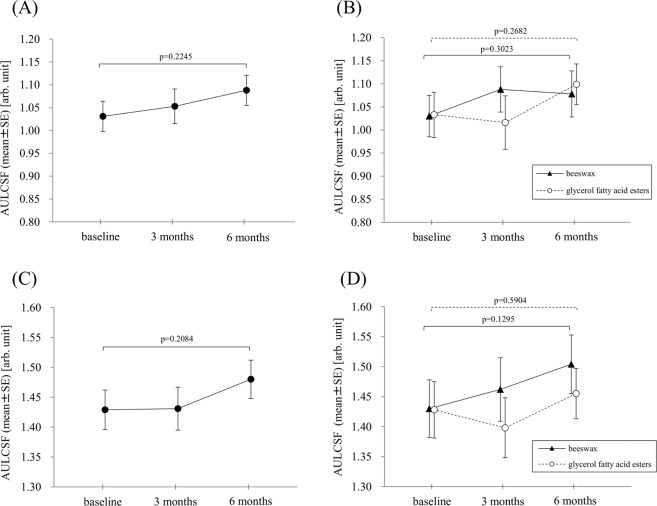
Area under log contrast sensitivity function (AULCSF) changes in the contrast glare test under the glare background (**A** and **B**) and mesopic background (**C** and **D**) in a total of 39 unilateral age-related macular degeneration patients (**A** and **C**) and the beeswax capsule group (n = 20) and glycerol fatty acid esters capsule group  (n = 19) (**B** and **D**). AULCSF did not significantly increase from the baseline in the total population and in each capsule group. SE = standard error.

Changes in the AULCSF under a glare background are shown in all 39 eyes, and AULCSF did not significantly increase between baseline and 3 months or 6 months (ANOVA, p = 0.2245) (Fig. 3A). Changes in AULCSF under a glare background in each capsule are shown in Fig. 3B. AULCSF in both capsule groups did not significantly increase from the baseline and 3 months or 6 months (ANOVA, p = 0.3023 in the beeswax capsule group and p = 0.2682 in the glycerol fatty acid esters capsule group).

Changes in AULCSF under a mesopic background are shown in all 39 eyes, and AULCSF did not significantly increase between the baseline and 3 months or 6 months (ANOVA, p = 0.2084) (Fig. 3C). Changes of AULCSF under a mesopic background in each capsule are shown in Fig. 3D. AULCSF in both capsule groups did not significantly increase from the baseline (ANOVA, p = 0.1295 in the beeswax capsule group and p = 0.5904 in the glycerol fatty acid esters capsule group).

### Sub-analysis: Factors associated with baseline plasma lutein concentration

#### Age, sex, and AMD subtype of fellow eyes

**Figure 4:**
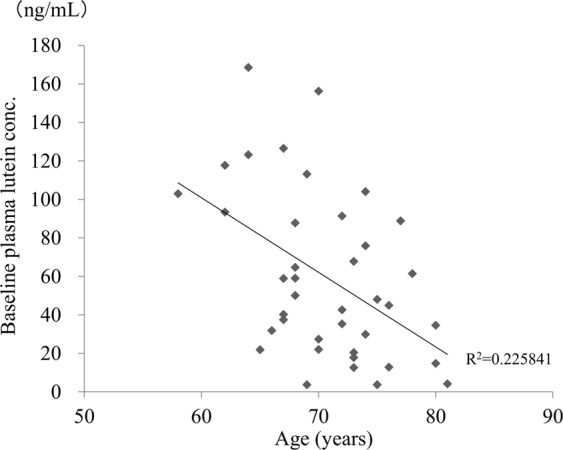
Scatter plot of age and baseline plasma lutein concentration. Baseline plasma lutein concentration = 333.41734 − 3.8749641 * age.

Plasma lutein concentration (±SE) at baseline (59.5 ± 6.9 ng/mL) was negatively correlated with age (correlation coefficient = −0.4469, p = 0.0043) (Fig. 4).

**Figure 5:**
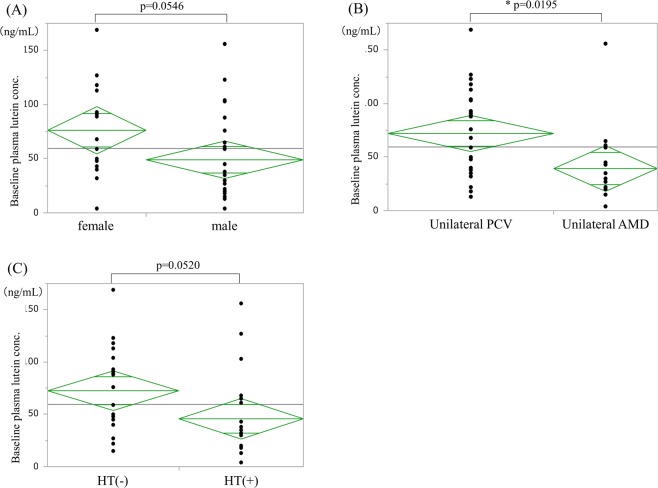
Composite figure demonstrating factors that decrease plasma lutein concentration. (**A**) Sex effects on baseline plasma lutein concentration. Baseline plasma lutein concentrations in males are lower than females (t-test, p = 0.0546). (**B**) AMD subtype in fellow eyes and baseline plasma lutein concentration. Baseline plasma lutein concentration in typical AMD patients was significantly lower than that in PCV patients (t-test, p = 0.0195). PCV = polypoidal choroidal vasculopathy. (**C**) Hypertension and baseline plasma lutein concentration. The plasma lutein concentration tended to differ in patients with or without hypertension. (t-test, p = 0.0520).

Regarding differences between the sexes, plasma lutein concentration at baseline was 49.0 ± 8.5 ng/mL in the 24 males and 76.3 ± 10.8 ng/mL in the 15 females, revealing a tendency toward a lower concentration in males (p = 0.0546) (Fig. 5A).

Based on the AMD subtype in fellow eyes, age (mean ± SD) was significantly different between typical AMD (15 patients, 73.5 ± 4.6 years old) and PCV (24 patients, 68.9 ± 5.0 years old) (p = 0.0065). Plasma lutein concentration (±SE) at baseline was 72.1 ± 8.3 ng/mL in 24 patients with unilateral PCV and 39.3 ± 10.5 ng/mL in 15 patients with unilateral typical AMD. Baseline plasma lutein concentration was significantly lower in patients with the typical AMD subtype (p = 0.0195) (Fig. 5B).

#### Hypertension

At baseline, mean plasma lutein concentration (±SE) was 72.6 ± 9.1 ng/mL in the 19 patients without hypertension (mean age: 69.6 ± 1.1 years) and 45.7 ± 9.8 ng/mL in the 20 patients with hypertension (mean age: 71.8 ± 1.2 years) (Fig. 5C). No significant difference of age was seen between these two groups; however, patients with hypertension showed a tendency to have lower plasma lutein levels (p = 0.0520).

#### Smoking

**Figure 6:**
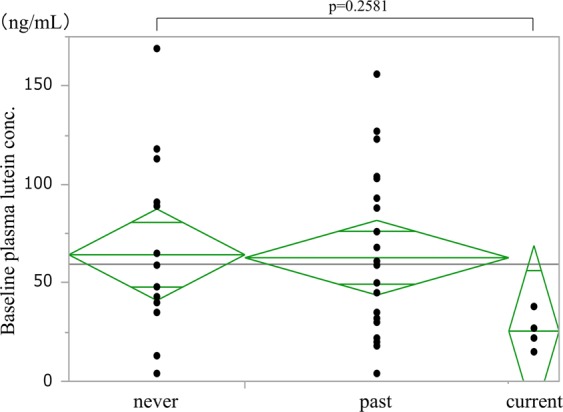
Smoking habit and baseline plasma lutein concentration. No significant difference was seen among the groups (ANOVA, p = 0.2581). The highest value in the current smoking group was lower than the mean value in the non-smoking and past smoking groups.

In assessments based on smoking history, mean plasma lutein concentration (±SD) at baseline was as follows: 64.3 ± 46.9 ng/mL in 14 never-smokers, 62.8 ± 43.1 ng/mL in 21 past smokers, and 25.5 ± 9.7 ng/mL in four current smokers (Fig. 6). No significant difference was seen among these groups (ANOVA, p = 0.2581).

Smoking index (ranging from 0 to 2650, mean (±SD) = 547 ± 606, median = 470) showed no significant correlation with plasma lutein concentration at baseline (Spearman’s rank correlation coefficient = −0.211, 95% confidence interval (CI): 0.112 to −0.494, p = 0.2026).

#### The frequency of green vegetable intake

**Figure 7:**
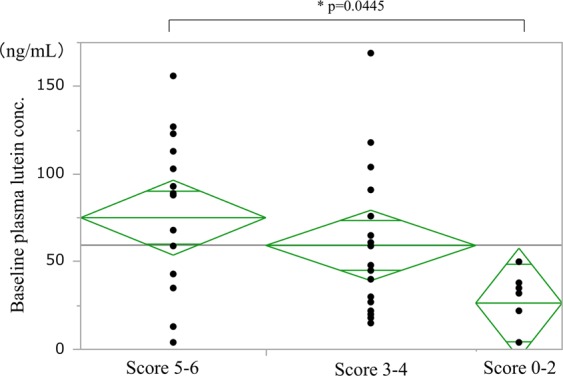
The frequency of green vegetable intake and baseline plasma lutein concentration. Score 0–2, equal to or less than one time in a week; score 3–4, 2–6 times in a week; score 5–6, at least one time in a day. A significant difference in baseline plasma lutein concentration was seen among the groups (ANOVA, p = 0.0445).

In the three groups, based on the frequency of green vegetable intake, the mean plasma lutein concentration was the highest (75.1 ng/ml) in the most frequent intake group (at least one time in a day; score, 5–6; n = 15). The lutein concentration was 59.3 ng/mL in the middle intake group (2–6 times in a week; score, 3–4; n = 17) and 26.4 ng/mL in the lowest intake group (equal to or less than one time in a week; score, 0–2; n = 7) (Fig. 7). A significant difference in plasma lutein concentration at baseline was seen among these three groups parallel to the frequencies (ANOVA, p = 0.0445).

#### A multiple regression analysis of associated factors and plasma lutein concentration at baseline

A multiple regression analysis including age, sex, type of AMD (typical AMD or PCV), hypertension, smoking status, and the frequency of green vegetable intake was conducted. This revealed that age (adjusted regression coefficient: −2.847, 95% CI: −5.406 to −0.288, p = 0.0305) was negatively associated with the plasma lutein concentration. In addition, consumption of green vegetable equal to or more than two times per day (score 6) was positively associated with the baseline plasma lutein concentration (adjusted regression coefficient: 52.111, 95% CI: 4.778 to 99.443, p = 0.0322) after adjusting for other factors.

## Discussion

The efficacy of 20-mg lutein supplementation in AMD patients undergoing anti-VEGF treatment by comparing two different capsules using either beeswax or glycerol fatty acid esters was investigated in this prospective, randomized, double-masked study. Although MPOD in the fellow eyes of unilateral AMD patients gradually increased from the baseline after 6 months’ continuous intake of the 20-mg lutein supplement of either capsule materials; however, MPOD did not significantly increase from the baseline. Plasma lutein concentration significantly increased to double the baseline value at 3 months and triple the baseline value at 6 months in all subjects. Over the first 3 months, the plasma lutein concentration in the beeswax capsule group increased more than that in the glycerol fatty acid esters capsule group. Although the capsule using glycerol fatty acid esters as a dispersant was developed to maximise dissolution, it was not more effective than the beeswax capsule in assessments based on MPOD measurements and plasma lutein concentration. Overall, the efficacy of the 20-mg lutein supplement over a 6-month period was elucidated in AMD patients in this study.

The quantitative assessment of lutein supplementation efficacy is limited by the need for special instruments for MPOD measurement or blood monitoring of plasma lutein concentration. Among these two parameters, plasma lutein concentration is believed to directly reflect the efficacy of lutein supplementation. Some previous studies showed that the blood lutein concentration increased 2–3-fold from the baseline in healthy subjects and patients with AMD and chronic central serous chorioretinopathy (CSC) after 20-mg lutein supplement intake^9,11,19^. However, measurement of blood lutein concentration is not a routine procedure because it requires a specialized HPLC examination. The subject number in the current study was only 39; however, monitoring of blood lutein concentration in actual AMD patients undergoing anti-VEGF therapy might yield precious data for clinicians to show the efficacy of lutein supplementation for AMD patients.

Several studies have monitored the macular pigment and contrast sensitivity following lutein intake, and these parameters generally increased after 20-mg lutein supplementation^21,22^. MPOD measurement using the autofluorescence method in affected eyes with AMD cannot provide exact values due to the autofluorescence abnormalities derived from exudate and choroidal neovascularization. Therefore, we obtained MPOD measurements in the fellow eyes of unilateral AMD patients, with the subject eyes being almost normal or showing early ARM with minimal autofluorescence abnormalities. In the current study, MPOD measured with the autofluorescence method in the fellow eyes of unilateral AMD patients showed a slight continuous increase at 3 and 6 months from the baseline. Although the overall increase in the MPOD value from the baseline to 6 months was very small (mean, 0.02 DU), this finding provides additional information regarding lutein supplementation efficacy in AMD patients. In addition, no significant increase was observed in contrast sensitivity under both mesopic and glare conditions, although a slight increasing tendency was observed at 6 months from the baseline. Additional studies of lutein supplement intake for a longer period are needed to examine the efficacy of MPOD and contrast sensitivity in AMD patients.

A sub-analysis of factors affecting the baseline plasma lutein concentration yielded some unexpected findings. Aging, smoking, hypertension, and a diet of less lutein-rich food are well-known risk factors for AMD that have been identified in a number of epidemiological studies^23–27^. Interestingly, in the current study, the baseline plasma lutein concentration showed a significant negative correlation with age, the presence of typical AMD in the fellow eye, and less frequent intake of green vegetables, and a negative tendency in males versus females and for patients with hypertension. Furthermore, a multiple regression analysis using these factors showed that age and green vegetable consumption were the only negative and positive factors, respectively, associated with plasma lutein concentration in AMD patients.

Baseline plasma lutein concentration significantly decreased with age in the current study and the mean value was 59.5 ng/mL, although its normal range or mean value is uncertain. In a previous study of 100 normal Japanese individuals with a mean age of 44.9 years, the mean plasma lutein concentration was 264 ng/mL (range, 101–569 ng/mL), with the concentration significantly increasing with age^8^. Another study on plasma lutein concentration in Japan reported a range of 70–80 ng/mL in patients with AMD or CSC^9,19^. Blood lutein concentration is thought to be strongly affected by diet; however, in comparisons of data obtained from the same country with similar diet patterns, a low plasma lutein level might be subsistent in patients with AMD or CSC. Long-term oxidative stress or unbalanced diet patterns and poorer gastric absorption in older patients are speculated to be the reasons underlying this finding.

In the current study, half of the patients had hypertension, and the mean plasma lutein concentration in patients with hypertension was approximately 60% of that in the patients without hypertension. As previously described, age is a negative factor for baseline plasma lutein concentration; however, the mean age was almost the same in the subjects with and without hypertension. Circulating carotenoid levels were inversely associated with hypertension in a previous report^28^. Thus, the levels of blood lutein, which is a carotenoid, may be deficient in AMD patients with hypertension beyond a certain age.

Regarding smoking status, the mean plasma lutein concentration in current smokers was less than half of that in never- and past smokers. The lack of a significant difference in this finding is likely due to the small number (four) of current smokers in this study. The maximum plasma lutein concentration in the current smoker group was 38 ng/mL, which was apparently lower than the mean plasma lutein concentration in the never- and past smokers (approximately 60 ng/mL). AMD in Asia is predominant in males because of a higher prevalence of smoking in this population^25^; in the current smoker group, all four patients were male. Smoking is a common problem associated with AMD worldwide^29,30^; however, the specific association between smoking and AMD is uncertain and is speculated to involve strong oxidative stress. Cessation of smoking is very important to prevent further AMD development. This action might have resulted in the increase in plasma lutein levels shown in the current study, although we could not show a clear correlation between smoking and plasma lutein concentration. However, these results may be used to encourage cessation of smoking in AMD patients as well as in young, healthy individuals.

The association between the frequency of green vegetable intake and plasma lutein concentration was interesting; mean plasma lutein concentration in the lowest intake group (equal to or less than one time in a week) was only one-third of that in the highest intake group (at least one time in a day). The two patients in the lowest intake group were taking warfarin and had restricted consumption of green vegetables with rich Vitamin K. As shown in a multiple regression model, the highest score of green vegetable consumption has positive association with higher plasma lutein concentration. High dietary consumption of lutein is associated with a lower incidence of AMD^31,32^. This result may provide hope for a potential intervention to prevent AMD. Our food-related questions were not specific, as shown in Table 1. However, the question regarding the frequency of green vegetable intake would be helpful to estimate the plasma lutein concentration level in AMD patients and to determine the need for lutein supplementation.

One patient discontinued this study due to the presence of yellow skin one month after starting the lutein supplement. That patient’s plasma lutein concentration at baseline was 199 ng/mL, which was the highest among the 42 subjects at enrolment. The patient had yellow skin as a result of excessive orange consumption in the past. However, the purpose of oral supplementation is to ‘supplement’ nutrients that are deficient in the patient. Therefore, while recommending lutein supplementation, clinicians should consider the baseline plasma lutein concentration, especially in patients with a history of yellow skin.

There are many limitations of this study. The number of participants in the current study was small (only 39 patients) and the study was performed at only one institute. The interviews for the frequency of green vegetable intake used non-specific questions. More specific nutritional investigations would have been preferable. Subjects in the AREDS2 were highly educated about AMD and were well-nourished^4^; however, subjects in the current study were not adequately educated about AMD, which might accurately represent patients in the real-world AMD clinics.

In conclusion, a significant increase of MPOD in the fellow eyes of unilateral AMD patients was not fully confirmed following 6 months intake of 20-mg lutein supplement. The beeswax capsule group seemed to have a slight increase in MPOD between 3 and 6 months, while glycerol fatty acid esters capsule group did not show increase in MPOD over 6 months. Given that plasma lutein concentration was significantly increased over the 6 months, it might take a longer duration to effect an increase in the MPOD. We also found that a higher consumption of green vegetable was positively associated with plasma lutein concentration after adjusting for other potential factors. Intake of green vegetable could therefore be a potential modifiable factor to prevent AMD.

## Supplementary information

Supplementary Information.

Supplementary Information2.

Supplementary Information3.

Supplementary Information4.

Supplementary Information5.

Supplementary Information6.

Supplementary Information7.

Supplementary Information8.
